# Revisiting the relationship between baseline risk and risk under treatment

**DOI:** 10.1186/1742-7622-6-1

**Published:** 2009-02-17

**Authors:** Hao Wang, Jean-Pierre Boissel, Patrice Nony

**Affiliations:** 1 Pharmacology Department, Shanghai Second Medical University, Shanghai, PR China; 2 Clinical Pharmacology Department, UMR CNRS 5558 Claude Bernard University Lyon 1, Cardiovascular Hospital, Lyon, France

## Abstract

**Background:**

In medical practice, it is generally accepted that the 'effect model' describing the relationship between baseline risk and risk under treatment is linear, i.e. 'relative risk' is constant. Absolute benefit is then proportional to a patient's baseline risk and the treatment is most effective among high-risk patients. Alternatively, the 'effect model' becomes curvilinear when 'odds ratio' is considered to be constant. However these two models are based on purely empirical considerations, and there is still no theoretical approach to support either the linear or the non-linear relation.

**Presentation of the hypothesis:**

From logistic and sigmoidal Emax (Hill) models, we derived a phenomenological model which includes the possibility of integrating both beneficial and harmful effects. Instead of a linear relation, our model suggests that the relationship is curvilinear i.e. the moderate-risk patients gain most from the treatment in opposition to those with low or high risk.

**Testing the hypothesis:**

Two approaches can be proposed to investigate in practice such a model. The retrospective one is to perform a meta-analysis of clinical trials with subgroups of patients including a great range of baseline risks. The prospective one is to perform a large clinical trial in which patients are recruited according to several prestratified diverse and high risk groups.

**Implications of the hypothesis:**

For the quantification of the treatment effect and considering such a model, the discrepancy between odds ratio and relative risk may be related not only to the level of risk under control conditions, but also to the characteristics of the dose-effect relation and the amount of dose administered. In the proposed approach, OR may be considered as constant in the whole range of *Rc*, and depending only on the intrinsic characteristics of the treatment. Therefore, OR should be preferred rather than RR to summarize information on treatment efficacy.

## Background

**Figure 1:**
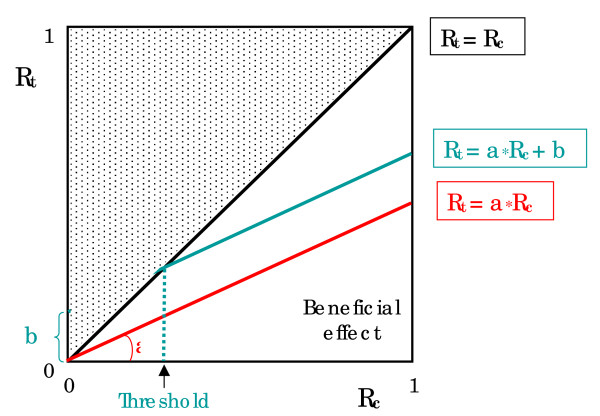
**L'Abbé plot showing the relationships between *Rc *(x axis) and *Rt *(y axis) presented in the condition that the 'effect model' is linear**. The identity line (*Rt *= *Rc*) corresponds with no treatment effect. Below this line, *Rt *is lower than *Rc *and the treatment is beneficial. Above this line the treatment is deleterious. A regression line may be estimated assuming the relative risk (RR) as a constant 'a' (Rt = a*Rc). In case of a full linear model (Rt = a*Rc + b), the treatment is beneficial for levels of *Rc *greater than the threshold, and deleterious for levels of *Rc *lower than the threshold. X axis: risk of event under control conditions (*Rc*), Y axis: risk of event under treatment (*Rt*).

The questions about whether and how the treatment benefit varies according to a patient's certain characteristics have deserved several works recently. [1-4] Available evidence as well as theoretical considerations support such links. Baseline risk (i.e. the risk of outcome for a patient under no treatment conditions) is used as a convenient summary of numerous characteristics which may be potential risk factors. The relation found between baseline risk and risk under treatment (quoted as the 'effect model' of the treatment [5]) indicates that some patients respond better, i.e. with a greater absolute risk reduction, to a given treatment than others do. L'Abbé plot [6] is a convenient graphical representation of the 'effect model', expressing on the x axis the risk of event under control conditions (*Rc*) and on the y axis the risk of event under treatment (*Rt*) (see Figure 1). The identity line corresponds with no treatment effect. For the dots below this line, *Rt *is lower than *Rc *and the treatment is beneficial. While for those falling above this line, the treatment seems unfavorable. A regression line may be estimated assuming the relative risk (RR) is constant, whereas absolute risk reduction (ARR) varies, possibly almost proportionately, with the baseline risk of patients. However, several problems accompany the linear relation between *Rc *and *Rt *: firstly, the linear model is purely empirical ; secondly, it does not take into account the range of variation of *Rc *and *Rt*, limited from 0 to 1 ; lastly, the validity of linear extrapolation for the patients in high risk (i.e. *Rc *> 0.5) remains most often unknown.

A simulation approach [7] based on numerical models of drug action has explored the whole range of *Rc *and suggested a curvilinearity at least in some settings. However, there is still no theoretical approach to support such a non-linear relation. Consequently, we propose an alternative approach to revisit the relationship between *Rc *and *Rt*, based on a phenomenological model that includes the possibility of integrating both beneficial and harmful therapeutic effects.

## Presentation of the hypothesis

First, let us assume that the probability (risk) of the outcome follows a logistic model [8]:

(1) $$\text{Ln}\left[\frac{R}{1-R}\right]=\beta_{0}+\beta_{1}\cdot \text{E}$$

Where *R *corresponds to the probability of the outcome, *β*_0 _and *β*_1 _are the intercept and slope of linear function respectively, and E is the pharmacodynamic effect (or similarly a risk factor of the spontaneous outcome).

Then let us suppose that the treatment affects E through a direct pharmacodynamic dose-response model (i.e. the sigmoidal Emax model or Hill model [9]) :

(2) $$\text{E }={\text{ E}}_{\text{0}}+\frac{{\text{E}}_{\text{max}}\cdot {\text{D}}^{\gamma }}{{\text{ED}}_{\text{50}}^{\gamma }+{\text{D}}^{\gamma }}$$

where E is the pharmacodynamic effect, E_0 _represents baseline value of E, E_max _is the maximum theoretical effect, D is the dose, ED_50 _is the dose at which 50% of the maximum effect is achieved, and *γ *is the sigmoidicity parameter.

Then, in treated subjects, the probability of outcome *Rt *and the treatment parameters are linked by :

(3) $$\text{Ln}\left[\frac{Rt}{1-Rt}\right]=\beta_{0}+\beta_{1}\cdot \left({\text{E}}_{0}+\frac{\text{Emax}\cdot {\text{D}}^{\gamma }}{{\text{ED}}_{\text{50}}^{\gamma }+{\text{D}}^{\gamma }}\right)$$

Letting D = 0 for the patients under control conditions, *Rc *is given by

(4) $$\text{Ln}\left[\frac{Rc}{1-Rc}\right]=\beta_{0}+\beta_{1}\cdot \left({\text{E}}_{0}\right)$$

Transforming equation (4) and substituting into (3), we have

(5) $$\text{Ln}\left[\frac{Rt}{1-Rt}\right]=\text{Ln}\left[\frac{Rc}{1-Rc}\right]+\beta \text{1}\cdot \frac{\text{Emax}\cdot {\text{D}}^{\gamma }}{{\text{ED}}_{50}^{\gamma }+{\text{D}}^{\gamma }}$$

a model with *Rc *as independent variable, *Rt *as dependent variable, and *β*_1_, E_max_, ED_50_, D, and *γ *as parameters.

Since $\frac{Rt/\left(1-Rt\right)}{Rc/\left(1-Rc\right)}$ is the odds ratio (OR) of the outcome, equation (5) becomes

(6) $$\text{Ln}\left[\text{OR}\right]=\beta_{\text{1}}\cdot \frac{{\text{E}}_{\text{max}}\cdot {\text{D}}^{\gamma }}{{\text{ED}}_{50}^{\gamma }{\text{+D}}^{\gamma }}$$

Therefore, OR which represents the size of treatment efficacy, does not vary with *Rc*. OR is here a constant only determined by pharmacotherapeutic parameters. This remains also true in case of an active control therapy (D ≠ 0).

**Figure 2:**
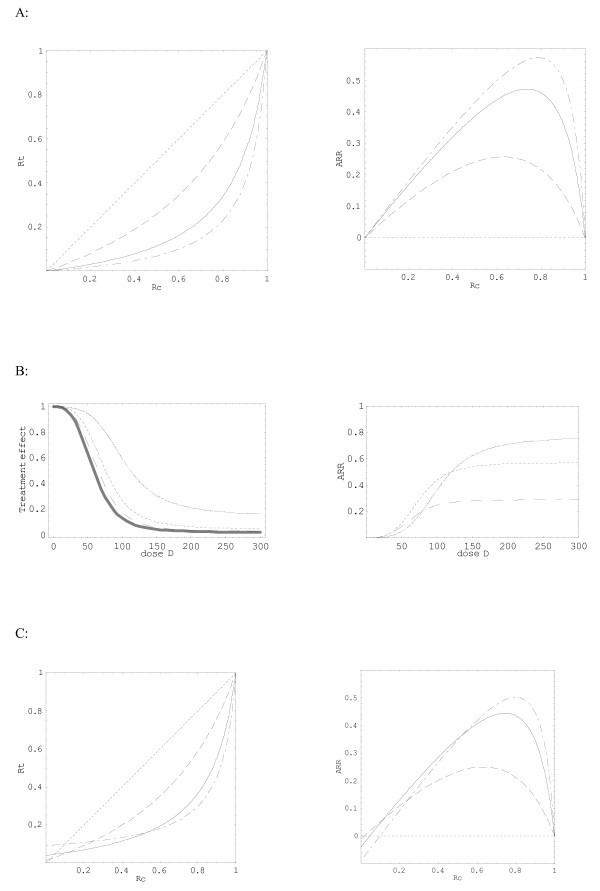
**A. Phenomenologically based simulation of the relationships between *Rc *and *Rt *(left column) or ARR (right column) presented in the condition that the treatment effect is beneficial**. The dotted line in the plots corresponds to the no effect line; the dash, solid, and dotted-dash line represent three different theoretical doses. The pharmacotherapeutic parameters, E_max_, ED_50_, *γ*, and *β*_1_, for beneficial effect, are -30, 100, 3, and 0.137 respectively. The three considered doses were fixed at 70, 100, and 120. X axis: risk of event under control conditions (*Rc*), Y axis: risk of event under treatment (*Rt*) (left column), absolute risk reduction (*ARR*), (right column). **B**. Phenomenologically based simulation of the relationships between *dose D *and *quantitative treatment effect *(left column) or ARR (right column) presented in the condition that the treatment effect is beneficial. The thick line corresponds to the relation between D and odds-ratio (OR) whatever the level of Rc, and the three thin lines to the relation between D and risk ratio (left column) or ARR (right column) for three levels of *Rc *(0.3, 0.6 and 0.9, dash, dotted and solid lines respectively). X axis: dose D, Y axis: OR or RR (thick or thin lines respectively) (left column), ARR (right column). **C**. Phenomenologically based simulation of the relationships between *Rc *and *Rt *(left column) or ARR (right column) presented in the condition that the treatment effect is biphasic. The dotted line in the plots corresponds to the no effect line; the dash, solid, and dotted-dash line represent three different theoretical doses. The pharmacotherapeutic parameters, E_max_, ED_50_, *γ*, and *β*_1_, for beneficial effect, are -30, 100, 3, and 0.137 respectively, and those for deleterious effect are 10, 250, 2, and 1.79 respectively. The baseline risk of harm (*Rc*_2_) is 0.0035. The three considered doses were fixed at 70, 100, and 120. X axis: risk of event under control conditions (*Rc*), Y axis: risk of event under treatment (*Rt*) (left column), absolute risk reduction (*ARR*), (right column).

Figure 2A shows the corresponding relations between *Rc *and *Rt *or ARR (left and right column respectively), assuming OR as a constant. In Figure 2B (left column), the quantitative treatment effect is shown as a function of the dose D. This effect is expressed either as an OR (thick line whatever the level of *Rc*) or a RR (thin lines for increasing levels of *Rc*). Compared with RR, the difference in the estimation of the treatment effect using OR is greater for high levels of *Rc*, and also depends on the value of the administered dose D. The same results are shown in Figure 2B (right column) using ARR as the expression of the quantitative treatment effect. Additional simulations could be performed considering the other parameters of the dose-effect relation (i.e. E_max_, ED_50 _and *γ*).

For a treatment with two independent mechanisms (one for the expected beneficial effect and the other for toxicity) and contributing to the same outcome (e.g. death), OR of benefit (OR_1_) or harm (OR_2_) can be expressed with its own pharmacological parameters as:

(7) $$\text{Ln}\left[{\text{OR}}_{i}\right]=\beta_{i}\cdot \frac{{\text{E}}_{\text{max-}i}\cdot {\text{D}}^{\gamma_{i}}}{{\text{ED}}_{\text{50-}i}^{\gamma_{i}}+{\text{D}}^{\gamma_{i}}}$$

where *i *= 1 for benefit and *i *= 2 for harm.

Since the joint probability of two independent events is calculated by *P *= *P*_1 _+ *P*_2 _- *P*_1_·*P*_2_, *Rt *and *Rc *can be written by :

(8) *Rt *= *Rt*_1 _+ *Rt*_2 _- *Rt*_1_·*Rt*_2 _and *Rc *= *Rc*_1 _+ *Rc*_2 _- *Rc*_1_·*Rc*_2_.

Then from models (7) and (8), for a treatment with one or two effects respectively, *Rt *and other measures of treatment effect, such as RR and ARR, can be easily translated and the relationships between baseline risk and treatment effect can be graphically simulated over the whole range of *Rc *(Figure 2C). The U-shape relation between *Rc *and *Rt *indicates that the patients with moderate risk obtain most from the treatment while those at highest risk benefit less or not at all. For a treatment involving two independent mechanisms, a harmful effect is observed in low-risk groups, and treatment effects under different doses show the potential difficulties of choosing an optimal dose for a patient, especially for those in low baseline risk.

## Testing the hypothesis

Two approaches (retrospective and prospective) can be proposed in order to investigate in practice such a non-linear 'effect model'. The retrospective one is to perform a meta-analysis of clinical trials with subgroups of patients with a great range of baseline risks. An alternative is to conduct a meta-analysis of individual patient data, a practical way of detecting differential treatment effects among various risk groups. [10] The prospective approach is to perform a large clinical trial ('megatrial') or several trials, in which patients are recruited according to several diverse risk groups that are prestratified depending on the information taken from previous small trials [11]. Severe patients, who are usually excluded from clinical trials, will be then preferentially included to detect the real treatment effect in high-risk conditions.

## Implications of the hypothesis

Using such a phenomenological approach based on the logistic and sigmoidal Emax (Hill) models, we observe a non-linear relation between *Rc *and *Rt*, that confirms previous results. [7,12]

These results are however strongly dependent on the underlying models considered. Logistic regression, one of a class of models known as generalized linear models, is a type of predictive model that can be used with two types of target variables : a categorical one that has exactly two categories (i.e., a *binary *or *dichotomous *variable), or a continuous one that has values in the range 0 to 1 representing probability values or proportions; explanatory variables can be either categorical or quantitative. The log odds is then a linear function of the explanatory variable(s), leading to an OR constant. This model is now used extensively in the medical science, where a clinical outcome is in most cases binary (e.g. death/alive, event/no event). The dose-concentration-effect model (e.g. Hill model) is based on the law of mass-action and on receptor occupancy theory, supposing a reversible drug pharmacodynamic effect which is related to the clinical (binary) outcome. Depending on the biological effects of drugs and their mechanisms of action, such assumptions may not be always valid, particularly in case of irreversible mechanisms, tolerance/rebound phenomena, and synergistic/antagonist effects.

For most patients (e.g. those in low or moderate risk), one can assume a linear relation with an absolute benefit proportional to the baseline risk. However for high-risk patients, appropriate data are required to show up whether it remains proportional or decreases to zero. The values of OR and RR may consequently differ according not only to the level of risk under control conditions (*Rc*), but also to the characteristics of the dose-effect relation and the amount of the dose chosen. In the proposed approach, OR may be considered as constant in the whole range of *Rc*, and depending only on the intrinsic characteristics of the treatment. Therefore, OR should be preferred to summarize information on treatment efficacy.

## Competing interests

The authors declared no conflict of interest and no funding source was involved in the creation of this manuscript.

## Authors' contributions

All authors declare that they participated in the conception and design of the phenomenological models, drafting the article and revising it critically and final approval of the version to be published.
